# Effects of Row-Type, Row-Spacing, Seeding Rate, Soil-Type, and Cultivar Differences on Soybean Seed Nutrition under US Mississippi Delta Conditions

**DOI:** 10.1371/journal.pone.0129913

**Published:** 2015-06-10

**Authors:** Nacer Bellaloui, Herbert A. Bruns, Hamed K. Abbas, Alemu Mengistu, Daniel K. Fisher, Krishna N. Reddy

**Affiliations:** 1 Crop Genetics Research Unit, USDA-ARS, Stoneville, MS, 38776, United States of America; 2 Crop Production Systems Research Unit, USDA-ARS, Stoneville, MS, 38776, United States of America; 3 Biological Control of Pests Research Unit, USDA-ARS, Stoneville, MS, 38776, United States of America; 4 Crop Genetics Research Unit, USDA-ARS, Jackson, TN, 38301, United States of America; University Paris South, FRANCE

## Abstract

The new Early Soybean Production System (ESPS), developed in the Midsouth USA, including the Mississippi delta, resulted in higher yield under irrigated and non-irrigated conditions. However, information on the effects of the agricultural practices such as row-type (RT: twin- vs. single-row), row-spacing, (RS), seeding rate (SR), soil-type (ST) on seed nutrition under the ESPS environment in the Mississippi delta is very limited. Our previous research in the Mississippi delta showed these agricultural practices altered seed nutrients in one cultivar only. However, whether these effects on seed nutrients will be exhibited by other soybean cultivars with earlier and later maturities across multiple years are not yet known. Therefore, the objective of this research was to evaluate the effects of agricultural practices and cultivar (Cv) differences on seed nutrition in clay and sandy soils under ESPS environment of high heat and drought. Two field experiments were conducted; one experiment was conducted in 2009 and 2010, and the other in 2008, 2009, and 2010 under irrigated conditions. Soybean were grown on 102 cm single-rows and on 25 cm twin-rows with 102 cm centers at seeding rates of 20, 30, 40, and 50 seeds m^–2^. Two soybean cultivars (94M80 with earlier maturity; and GP 533 with later maturity) were used. Results showed that increasing seeding rate resulted in increases of protein, sucrose, glucose, raffinose, B, and P concentrations on both single- and twin-rows. However, this increase became either constant or declined at the higher rates (40 and 50 seeds m^–2^). Protein and linolenic acid concentrations were higher in GP 533 than in 94M80 on both row-types, but oil and oleic acid concentrations were in 94M80 than GP 533. Generally, cultivar GP 533 accumulated more seed constituents in seeds than 94M80. In 2010, there were no clear responses of seed nutrients to SR increase in both cultivars, perhaps due to drier year and high heat in 2010. It is concluded that RT and SR can alter seed nutrition under clay and sandy soils, especially under high heat and drought conditions as in the Mississippi delta.

## Introduction

Soybean is a major crop in the world, and its seed is an important source of protein and oil. On seed dry weight-bases, seed protein ranges from 38 to 42% and oil from 19–22%. Soybean oil includes saturated fatty acids, such as palmitic (12–13% of total oil) and stearic (3–4%), and unsaturated fatty acids such as oleic (19–23%), linoleic (48–58%), and linolenic (5–8%). Soybean seed also contains sugars, including monosaccharides (glucose and fructose), disaccharide (sucrose), and oligosaccharides (raffinose and stachyose). Soybean seed is an important source of minerals such as K, Ca, Zn, Fe, B, and P. High oleic acid and low linolenic acid are desirable for the oil industry because of their contributions to oil stability and shelf life. Similarly, high glucose, fructose, and sucrose levels are desirable because of their contributions to taste and flavor of seed, but high raffinose and stachyose levels are undesirable because they are indigestible and cause flatulence or diarrhea in non-ruminant animals such as chickens and pig [1]. Although seed composition constituents are genetically controlled, they are known to be influenced by genotype, growing season, geographic location, and agronomic practices (Wilcox and [2, 3, 4, 5]. For example, The interrelationships between seed protein, oil, and oligosaccharides (sucrose, raffinose, and stachyose) were determined under three different environments conditions in forty-three breeding lines that widely varied in seed protein concentration(from 413 to 468 g kg^-1^) [2]. It was found that both environment and breeding lines had significant effects of these traits, and the increases in protein concentrations occurred at the expenses of oil and carbohydrates. They concluded that decreases in carbohydrates resulted in increases in protein, resulting in higher nutritional value of the meal [2]. Also, it was found that soybean genotypes grown in various locations in Missouri and Illinois in USA showed significant differences in oil, protein, and fatty acid composition, and soybean grown on irrigated land resulted in higher polyunsaturated fatty acids (for example, linoleic and linolenic acids) and lower in iron contents compared to those grown on nonirrigated land [5]. They concluded that the variability in crude oil composition in soybean grown on different types of soils under different climatic conditions requires much more efforts to understand the mechanisms of these changes. Biosynthesis of oil depends on the enzymes involved, nutrients absorbed from soils that contribute to their enzyme activity, and environmental factors [5]. The interaction of all these factors and how they influence agricultural practices are still not yet understood, and need in-depth research to understand this divergence in seed composition constituents [5]. Therefore, optimizing agricultural practices with variable environmental factors, especially temperature, drought, and soil conditions, is vital to maintain high nutritional seed quality.

Although the early Soybean Production System (ESPS) in the Midsouth US resulted in higher yield [6, 7] under irrigated and non-irrigated conditions, poor seed quality is still a concern [8]. It was indicated that seed produced under high-temperature and high-humidity production environments such as in ESPS is prone to seed quality issues [8]. Under the ESPS conditions, growing soybean on twin rows became popular due to higher yield compared with single-row [9, 10], and the use of raised beds permitted the use of furrow irrigation [9, 11].

Currently, there is limited information available on the effects single- vs. twin-row planting and seeding rates on seed nutrition in soybean in the Mississippi delta in clay and sandy soils. Even the limited information available on the effect of row-spacing and seeding rates on seed nutrition is still inconsistent. For example, the effect of row-spacing (RS) and irrigation on soybean seed protein, oil, and fatty acids was studied and found that row-spacing and irrigation significantly affected protein and oil contents, and that RS of 70 cm resulted in the highest protein content, followed by RS of 60, 40, and 50 cm [12]. They also found that RS had a significant (P < 0.01) influence on oleic and linoleic acid contents, and that a row-spacing of 50 cm produced maximum oil, while a row-spacing of 70 cm produced the highest protein (39.05%), and 50 cm produced the lowest protein (37.65%). They also found that oil and protein contents were both affected by year, and protein and oil content were inversely correlated (r = -0.791 in 1998 and -0.721 in 1999). Recently, the effect of row-spacing (38 and 76 cm) and seeding rate (from 247,000 to 592,800 seeds ha^-1^ for cultivars P 93M90 and AG 3906, and from 60,000 to 180,000 seeds ha^-1^ for cultivar V 52N3 and P 94B73) was evaluated and found that seed protein, oleic acid, sugars, P, and B concentrations increased with the increase of seeding rate in P 93M90 and AG 3906 [13]. However, after the maximum concentrations of these constituents were reached, a decrease was observed at the highest seeding rate. They also found that this trend was only observed in 2006, and was depended on row-spacing. In 2007, protein and oleic acid concentrations decreased, opposing the trend in 2006, indicating environmental factor effects such as heat and drought on trend of seed nutrients. In cultivars P 94B73 and V 52N3 seed protein concentration increased with seeding rate in 2006 and 2007 for both 38 and 76 cm row-spacing (Bellaloui et al., 2014). Research in this area on other species revealed that there were no consistent effects of seeding rate on seed nutrition in rapeseed (*Brassica napus* L.) [14], and the effects of seeding rate and row-spacing on oil and protein concentration were highly variable. Other researchers investigated the effects of three row-spacing of 30, 40, and 50 cm on canola, and found that there were no relationship between row-spacing and oil in canola, but interactions between varieties and row-spacing were observed, with the highest oil concentration recorded at row-spacing of 30 cm [15]. Also, sesame seed protein, total oil, and fatty acids were found to be influenced by row-spacing and irrigation, and found that oil and protein contents were significantly different among treatments [16]. They found that protein content was significantly influenced by row-spacing and irrigation, and that row-spacing of 70 cm had the highest protein followed by row-spacing of 60, 50, and 40 cm, and the highest oleic acid was observed in row-spacing of 70 cm, while the highest linoleic acid was observed in row-spacing of 40 cm. The different responses of protein, oil, and fatty acids to row-spacing were reported to be dependent on environmental conditions in each year, especially temperature and rainfall [13, 17, 18, 19].

Based on the above discussion, it is clear that although the ESPS resulted in higher yield under irrigated and non-irrigated, the effects of agricultural practices such as twin- vs. single-row planting, seeding rate, and row-spacing on seed nutrition status under the new environment of the ESPS under Mississippi delta conditions has not been well investigated. Therefore, the objective of the current research was to evaluate the effects of twin- vs. single-row planting, seeding rate, and row-spacing on seed protein, oil, fatty acids, sugars, and minerals in two row-spacing (38 and 76 cm) in clay and sandy soils. To investigate the possible different response of seed nutrition in different cultivars, two soybean cultivars (94M80 and GP 533) belong to different maturity groups (earlier and later maturities) were used. The current research would allow optimizing agricultural practices under variable environmental factors of high heat, drought, and soil conditions in the ESPS and maintain high nutritional seed quality.

## Materials and Methods

### Field management and growth conditions

A field experiment was conducted at Stoneville, MS, in Sharkey clay soil in 2009 and 2010, and in Beulah fine sandy loam soil in 2008, 2009, and 2010. This research was a part of a larger research project that involved different research components. We focus here only on the seed nutrition quality component; however, the agronomic component, including yield, was reported [9] where a detailed description of field management and growth conditions was provided. Briefly, the sites were prepared each year by disking and bedding in the fall of the previous year. Raised beds spaced 102 cm apart were formed with a bedding hipper. Prior to planting, raised beds were smoothed with a harrow as needed to plant soybean in a 102 cm single-row and 25-cm twin rows on 102-cm centers and to facilitate furrow irrigation. Cultivar Armor GP 533 (Armor Seed Company, Waldenburg, AR), a mid-MG V [20] and Pioneer 94M80 (Pioneer Hi-Bred Int., Huntsville, AL), a late MG IV, were used. Each plot was four-rows wide, planted 11 m long, and end-trimmed to 9 m at the V4 growth stage. Single-row plantings were conducted using an Almaco cone plot planter and twin-row plots were planted using a four unit Monosem NG-3 twin-row vacuum planter set on 102 cm centers between planting units and 25 cm between rows within a unit. In the sandy loam soil, planting dates were 16 April in 2008, 18 April in 2009, and 14 April in 2010. On the Sharkey clay site, planting dates were 22 April in 2009 and 12 April in 2010. Weed control in both sites was achieved by applying a pre-plant application of trifluralin [2,6-Dinitro-*N*,*N*-dipropyl-4-(trifluoromethyl)aniline] at 0.7 kg ai ha^–1^ followed by two postemergence applications of metolachlor [2-chloro-N-(2-ethyl-6-methylphenyl)-N-(2-methoxy- 1-methylethyl) acetamide] and glyphosate [2-[(phosphonomethyl)amino]acetic acid] at growth stage V2 to V3 (two to three trifoliate) and at V5 to V6 (five to six trifoliate). For fungal control, pyraclostrobin (carbamic acid, [[[1-(4-cholrophenyl)-H-pyrazol-3-yl]oxy]methyl]phenyl]methoxy-,methyl ester) was applied according to factory label directions at V5 to V6. Plots were furrow-irrigated from R1 (beginning flowering) through R6 (full seed-fill) with approximately 25 mm water applied at 10 day intervals. Seeds at maturity harvest (R8 stage) were collected and processed, and nutrients were determined as described below. All samples (seeds, leaves, and soil) were processed after sampling and harvesting in a similar way in each year and measured with the same instruments under similar conditions to minimize the variability due to instruments and measurement conditions.

The authority responsible for the field experiments that allowed this research activity is the United States Department of Agriculture, Agricultural Research Services (USDA-ARS), Stoneville, MS. This research activity was conducted under the authority of USDA-ARS, and this study does not require any specific permission because it is owned by USDA-ARS for routine research activities. This research did not involve endangered or protected species.

### Soil minerals, N, S, and C analysis

Analysis of minerals K, P, B, and Fe, and N, S, and C in soils was conducted by the University of Georgia’s Soil, Plant, and Water Laboratory, Athens, GA. The concentration of K was determined using a 5 g soil: 20 ml Mehlich-1 solution and analyzed using inductively coupled plasma spectrometry (Thermo Jarrell-Ash Model 61E ICP and Thermo Jarrell-Ash Autosampler 300). Percentages of N, S, and C were determined on a 0.25 g sample of soil by combusting samples in an oxygen atmosphere at 1350°C, converting elemental N, S, and C into N_2_, SO_2_, and CO_2_, respectively. Then, these gases were passed through infrared cells and N, S, and C were determined by an elemental analyzer using thermal conductivity cells (LECOCNS-2000 elemental analyzer, LECO Corporation, St. Joseph, MI, USA) [13, 21].

### Leaf and seed minerals, N, S, and C analysis

Leaf and seed samples were analyzed for minerals, and N, S, and C by digesting 0.6 g of dried, ground plant materials in HNO_3_ in a microwave digestion system. Samples were ground using a Laboratory Mill 3600 (Perten, Springfield, IL, USA), and the concentration of K was determined using inductively coupled plasma spectrometry [13, 21]. For N, C, and S measurements, a 0.25 g ground-dried sample was combusted in an oxygen atmosphere at 1350°C, converting elemental N, S, and C into N_2_, SO_2_, and CO_2_, respectively. These gases were then passed through infrared cells and N, S, and C are determined by an elemental analyzer using thermal conductivity cells (LECOCNS-2000 elemental analyzer, LECO Corporation, St. Joseph, MI USA) [13, 21].

### Seed analysis for protein, oil, and fatty acids

Mature seeds at the R8 stage were collected and analyzed for protein, oil, and fatty acids. Samples of 25 g of seed were ground using the Laboratory Mill 3600 and analyzed by near infrared reflectance [2, 13, 18] using a diode array feed analyzer AD 7200 (Perten, Springfield, IL USA). An initial calibration equation was developed by the University of Minnesota using Preteen’s Thermo Galactic Grams PLS IQ software, and the calibration curve was established using conventional chemical protocols, using AOAC methods [22, 23]. Protein and oil concentrations were determined based on a seed dry matter [2, 13, 13]. The concentrations of palmitic, stearic, oleic, linoleic, and linolenic fatty acids were determined on a total oil basis [13].

### Seed analysis for sucrose, raffinose, and stachyose

Seed samples at the R8 stage were collected for sugar analysis. Seed samples of 25 g from each plot were ground using the Laboratory Mill 3600. Determination of seed sugars was conducted by near infrared reflectance [2, 13] using the AD 7200 array feed analyzer. The analyses of sugars were based on a seed dry matter basis [2, 12, 13].

### Glucose determination in seed

Glucose concentration in seed was determined by an enzymatic reaction using a Glucose (HK) Assay Kit, Product Code GAHK-20 (Sigma-Aldrich Co, St Louis, MO USA) as detailed elsewhere [13]. Glucose concentrations were determined by reading samples at absorbance of 340 nm using the Beckman Coulter DU 800 spectrophotometer, with concentrations of glucose expressed as mg g^-1^ dry weight.

### Fructose determination in seed

Fructose concentration in seed was determined based on an enzymatic reaction using a Fructose Assay Kit, Product Code FA-20 (Sigma-Aldrich Co., St. Louis, MO, USA) as described elsewhere [13]. Fructose concentration was determined by the Beckman Coulter DU 800 spectrophotometer and by reading the absorbance of samples at 340 nm, with fructose concentrations in seeds expressed as mg g^-1^ dry weight.

### Boron determination

Boron concentration in leaves and seeds was determined using the Azomethine-H method [13, 24, 25] as reported elsewhere [13]. Briefly, a sample of 1.0 g was ashed at 500°C and extracted with 20 ml of 2 M HCl at 90°C for 10 minutes. A 2-ml sample of the filtered mixture was then added to 4 ml of buffer solution (containing 25% ammonium acetate, 1.5% EDTA, and 12.5% acetic acid). Four ml of fresh azomethine-H solution (0.45% azomethine-H and 1% of ascorbic acid) [26] was added. The concentration of boron in leaves and seeds was determined by reading the samples at 420 nm using a Beckman Coulter DU 800 spectrophotometer (Beckman Coulter, Inc., Brea, CA, USA).

### Iron determination

Concentrations of Fe in leaves and seeds were determined according to the methods described elsewhere [27, 28] by acid wet digestion, extraction, and reaction of the reduced ferrous Fe with 1,10-phenanthrolineand, and as described elsewhere [13]. Briefly, a random sample of 2 g of dried ground leaves or seeds were acid digested, and the soluble constituents were dissolved in 2 M HCl. A volume of 4 ml of an aliquot containing 1–20 μg of iron of the sample solution was transferred into a 25-ml volumetric flask and diluted to 5 ml using 0.4 M HCl. One ml of quinol solution was added to the 5 ml diluted sample solution and mixed, and 3 ml of the phenanthroline solution and 5 ml of the tri-sodium citrate solution (8% w/v) were added. After the solution was diluted to 25 ml with distilled water, the solution was incubated at room temperature for 4 h. Phenanthroline solution of 0.25% (w/v) in 25% (v/v) ethanol and quinol solution (1% w/v) reagent was prepared. Fresh standard solutions of Fe ion concentrations in 0.4 M HCl, ranging from 0.0 to 4 μg ml^−1^ of Fe, was prepared to establish standard curves. Iron concentrations in the samples were determined by reading the samples at absorbance of 510 nm using the Beckman Coulter DU 800 spectrophotometer.

### Phosphorus determination

The concentrations of P in leaves and seeds were determined based on the yellow phosphor-vanado-molybdate complex according to others [29], and as described in details by others [13]. A sample of 2 g dried, ground leaf or seed sample was ashed, and 10 ml of 6 M HCl was added. The samples were placed in a water bath at 100°C to evaporate the solution to dryness. After the extraction of P using 2 ml of 36% v/v HCl under heat and filtration, 5 ml of 5 M HCl and 5 ml of ammonium molybdate–ammonium metavanadate reagent were added to 5 ml of the filtrate. Ammonium molybdate–ammonium metavanadate was prepared in 500 ml of distilled water by dissolving 25 g of ammonium molybdate and 1.25 g of ammonium metavanadate. A phosphorus standard curve was established by preparing standard solutions of P concentrations ranging from 0–50 μg ml^−1^ using dihydrogen orthophosphates. Phosphorus concentrations were determined by reading the absorbance at 400 nm using the Beckman Coulter DU 800 spectrophotometer.

### Experimental design and statistical analysis

The experimental design was a split-plot in a randomized complete block, with cultivar as a main plot and a combination of row type (either a single- or twin-row planting) and seeding rates (20, 30, 40, or 50 seeds m^–2^) as subplots. Four replicates were used, with replicate (year) and seeding rate × replicate (year) considered as components of variance for random effects. Year, cultivar, and seeding rate were modeled as fixed effects. Estimated residuals of random effect factors as covariance parameters were indicated in tables. Residual values in tables refer to Restricted Maximum Residual Likelihood (REML), which reflects the total variance of the random parameters in the model. Analysis of variance of data was conducted using PROC MIXED in SAS [30]. Means were separated by Fisher’s protected LSD (0.05). Correlation between seeding rates and seed constituents was performed using PROC CORR in SAS.

## Results

### Levels of nutrients in soil and leaf tissues

Analyses during the vegetative stages of soybean at both sites showed no nutrient deficiencies were observed in soils in 2009 and 2010. Briefly, in 2009 and 2010, respectively, the average nutrient levels in the clay soil were C = 1.2 and 1.5%; N = 0.10 and 0.13%; S = 0.30 and 0.53%; K = 2.76 and 2.24 g kg^-1^; P = 327 and 321 mg kg^-1^; B = 2.03 and 2.13 mg kg^-1^; and Fe = 18.33 and 20.45 g kg^-1^. In the sandy soil, the average nutrient levels in 2009 and 2010, respectively were: C = 1.01 and 1.14%; N = 0.09 and 0.13%; S = 0.25 and 0.29%; K = 2.34 and 2.13 g kg^-1^; P = 233 and 217 mg kg^-1^; B = 1.10 and 1.35 mg kg^-1^; and Fe = 19.45 and 21.54 g kg^-1^. Analysis of random samples of the fully expanded leaves at the R5-R6 stages showed that average nutrient concentrations in leaves at both sites were adequate; however, the concentrations of nutrients in 2009 were higher than in 2010 due to high heat and a drier year in 2010, especially for K, B, P, and Fe. The concentrations of nutrients in leaves in the clay soil, respectively in 2009 and 2010 were: N = 5.11 and 4.02%; S = 0.25 and 0.19%; K = 2.3 and 1.34%; P = 0.42 and 0.29%; B = 45.12 and 30.34 mg kg^-1^; and Fe = 232 and 96 mg kg^-1^. In the sandy soil, the nutrient levels in leaves were N = 5.4 and 4.32%; S = 0.31 and 0.29%; K = 2.7 and 1.52%; P = 0.37 and 0.25%; B = 38.11 and 30.51 mg kg^-1^; and Fe 184 and 146 mg kg^-1^.

### Analysis of variance for seed protein, oil, and fatty acids

In the clay soil, ANOVA analysis (Table 1) showed that year (Y) and cultivar (Cv) had significant (P≤0.05) effects on protein, oil, oleic, and linolenic acid concentrations, indicating the importance of environmental factors in each year and cultivar influences on some seed constituents. The significant effects of row type (single- or twin-row) on protein, oil, palmitic, and linolenic acid concentrations, and the effects of seeding rate (SR) on protein and palmitic acid, and the effects of Y × Cv × SR interactions on protein and stearic acid indicated the influence of agricultural practice such as RT and SR on seed nutrition. The significant effects of Y × Cv × RT interactions for protein, oleic, linoleic, and linolenic acid concentrations showed that the influence of Cv and RT on seed composition were dependent on the environmental conditions of that year.

**Table 1:** Analysis of variance (*F* and *P* values) of the effects of year (Y), cultivar (Cv), row-type (RT, either single- or twin-row), and seeding rate (SR) on the concentrations of seed protein, oil, and fatty acids (g kg^-1^) in soybean cultivars 94M80 and GP 533 in Sharkey clay soil.^a^

		Protein		Oil		Palmitic		Stearic		Oleic		Linoleic		Linolenic	
Effect	DF	*F*	*P*	*F*	*P*	*F*	*P*	*F*	*P*	*F*	*P*	*F*	*P*	*F*	*P*
**Y**	1	325	***	206	***	0.78	NS	309	***	117	***	3.46	NS	200	***
**Cv**	1	217	***	191	***	51.8	***	2.10	NS	729	***	28.3	***	355	***
**Y × Cv**	1	12.7	***	63.2	***	7.41	**	28.5	***	0.48	NS	28.5	***	122	***
**RT**	1	22.6	***	326	***	6.08	*	0.56	NS	NS	NS	2.02	NS	11.2	***
**Y × RT**	1	0.01	NS^‡^	2.77	NS	0.11	NS	0.91	NS	0.03	NS	13.5	***	13.7	***
**Cv × RT**	1	1.18	NS	39.9	***	0.56	NS	1.47	NS	0.90	NS	6.09	*	15.6	***
**Y × Cv × RT**	1	5.95	*	1.90	NS	0.03	NS	0.88	NS	6.26	*	15.8	***	13.5	***
**SR**	3	7.31	***	1.12	NS	3.84	**	0.11	NS	0.38	NS	0.49	NS	1.41	NS
**Y × SR**	3	12.0	***	0.88	NS	2.16	NS	1.07	NS	0.07	NS	1.28	NS	2.39	NS
**Cv × SR**	3	2.69	*	0.52	NS	2.23	NS	2.96	*	0.12	NS	1.44	NS	0.21	NS
**Y × Cv × SR**	3	3.17	*	0.62	NS	0.95	NS	3.84	**	0.15	NS	0.99	NS	1.80	NS
**RT × SR**	3	5.44	**	2.65	NS	0.67	NS	0.21	NS	0.37	NS	0.25	NS	1.31	NS
**Y × RT × SR**	3	4.66	**	0.31	NS	0.54	NS	1.17	NS	0.68	NS	0.40	NS	0.26	NS
**Cv × RT × SR**	3	1.73	NS	1.47	NS	0.05	NS	2.72	*	0.35	NS	0.04	NS	0.77	NS
**Y × Cv × RT × SR**	3	1.12	NS	1.47	NS	0.99	NS	0.16	NS	0.57	NS	1.23	NS	1.42	NS
**Residual**		26.7		13.4		35.0		1.1		103		145		52.3	

The experiment was conducted in 2009 and 2010 in Stoneville, MS, USA.

^a^ *Significant at *P* ≤ 0.05; **Significant at *P* ≤ 0.01; ***Significant at *P* ≤ 0.001.

^‡^NS, not significant.

In the sandy loam soil, ANOVA (Table 2) also showed a significant influence of Y, Cv, and SR on seed protein, palmitic, stearic, oleic, and linolenic acid concentrations. The interaction of Y × Cv × SR was not significant for protein, but Cv × SR was significant, and the significant effects of the former is due to the presence of year in the model of Cv × SR, introducing a new growing environment. Seed constituents responded differently to similar factors, depending on soil texture and nutrient content. For example, Y and Cv had significant effects on protein, oil, oleic, and linolenic acids in clay soil, but Y and Cv had significant effects on protein, palmitic, stearic, oleic, and linolenic acids in sandy soil, indicating the significant influence of soil texture and nutrient content (Table 1 and Table 2).

**Table 2:** Analysis of variance (*F* and *P* values) of the effects of year (Y), cultivar (Cv), row-type (RT, either single- or twin-row), and seeding rate (SR) on the concentrations of seed protein, oil, and fatty acids (g kg^-1^) in soybean cultivars 94M80 and GP 533 in Beulah fine sandy loam soil. ^a^

		Protein		Oil		Palmitic		Stearic		Oleic		Linoleic		Linolenic	
Effect	DF	*F*	*P*	*F*	*P*	*F*	*P*	*F*	*P*	*F*	*P*	*F*	*P*	*F*	*P*
**Y**	2	58.1	***	61.4	***	19.4	***	73.7	***	104	***	3.41	NS	167	***
**Cv**	1	639	***	1431	***	72.2	***	20.4	***	333	***	36.9	***	158	***
**Y × Cv**	2	28.1	***	19.4	***	31.2	***	19.2	***	58.0	***	6.64	**	78.0	***
**RT**	1	28.7	***	0.21	NS	0.45	NS	1.38	NS	2.6	NS	0.66	NS	0.45	NS
**Y × RT**	2	10.8	***	29.0	***	0.50	NS	0.0	NS	0.1	NS	0.0	NS	0.03	NS
**Cv × RT**	1	17.3	***	12.5	***	0.81	NS	0.71	NS	0.0	NS	0.06	NS	1.10	NS
**Y × Cv × RT**	2	12.4	***	2.65	NS	0.46	NS	0.13	NS	1.85	NS	0.8	NS	0.24	NS
**SR**	3	6.01	***	2.48	NS	6.41	***	3.24	*	9.95	***	1.01	NS	15.1	***
**Y × SR**	6	2.88	*	3.29	*	7.7	***	0.8	NS	7.0	***	1.37	NS	2.72	*
**Cv × SR**	3	6.87	***	1.89	NS	2.18	NS	0.63	NS	1.85	NS	1.29	NS	4.68	**
**Y × Cv × SR**	6	1.27	NS^‡^	2.69	*	1.98	NS	0.31	NS	1.26	NS	0.26	NS	3.19	**
**RT × SR**	3	2.90	*	5.55	***	0.10	NS	2.09	NS	0.96	NS	2.22	NS	1.24	NS
**Y × RT × SR**	6	1.01	NS	5.82	***	1.15	NS	2.9	*	0.09	NS	0.61	NS	0.79	NS
**Cv × RT × SR**	3	1.61	NS	10.3	***	0.94	NS	0.21	NS	0.59	NS	0.21	NS	1.98	NS
**Y × Cv ×**	6	9.14	***	5.35	**	0.31	NS	1.23	NS	0.90	NS	0.5	NS	1.28	NS
**RT×SR**															
**Residual**		47.9		20.7		32.6		1.1		176		267		40	

The experiment was conducted in 2008, 2009, and 2010 in Stoneville, MS, USA.

^a^ *Significant at *P* ≤ 0.05; **Significant at *P* ≤ 0.01; ***Significant at *P* ≤ 0.001.

^‡^NS, not significant.

### Analysis of variance for seed sugars and minerals

In the clay soil, there were significant (P≤0.05) influences of Cv, RT, and SR on sucrose, glucose, fructose, and B concentrations (Table 3). There were no significant effects of Y on seed B, P, and Fe or seed sugars, except for glucose concentrations. The response of sucrose or glucose concentrations to RT or SR depended on Y and Cv, as indicated by the significant interactions between these factors. The sugars that the least affected by these factors were raffinose and stachyose, and the more responsive sugars were sucrose (disaccharide) and glucose (monosaccharide). In the sandy soil, Y, RT, and SR had significant effects on sucrose, glucose, fructose, and B and P concentrations (Table 4). The cultivar had significant effects on minerals (B, P, and Fe) and sugars, except sucrose, indicating the different response of cultivars to agricultural practices for sugars.

**Table 3:** Analysis of variance (*F* and *P* values) of the effects of year (Y), cultivar (Cv), row-type (RT, either single- or twin-row), and seeding rate (SR) on the concentrations of seed sugars (sucrose, raffinose, stachyose, glucose, and fructose, mg g^-1^) and boron (B, mg kg^-1^), phosphorus (P, g kg^-1^), and iron (Fe, mg kg^-1^) in soybean cultivars 94M80 and GP 533 in Sharkey clay soil. ^a^

		Suc		Raff		Stac		Glu		Fruc		B		P		Fe	
Effect	DF	*F*	*P*	*F*	*P*	*F*	*P*	*F*	*P*	*F*	*P*	*F*	*P*	*F*	*P*	*F*	*P*
**Y**	1	0	NS	0.0	NS	0.0	NS	819	***	0.0	NS	0.0	NS	0.0	NS	0.0	NS
**Cv**	1	374	***	4036	***	1672	***	94.9	***	96	***	313	***	82.5	***	531	***
**Y × Cv**	1	0.00	NS	0.0	NS	0.0	NS	63.6	***	0.0	NS	0.0	NS	0.0	NS	0.0	NS
**RT**	1	25.5	***	0.04	NS	1.46	NS	23.6	***	158	***	71.2	***	510	***	646	***
**Y × RT**	1	0.0	NS	0.0	NS	0.00	NS	15.8	***	0.0	NS	0.0	NS	0.0	NS	0.0	NS
**C × RT**	1	30.6	***	1.72	NS	3.15	NS	19.0	***	4.53	*	14.9	***	47.24	***	0.21	NS
**Y × Cv × RT**	1	0.0	NS	0.0	NS	0.0	NS	12.7	***	0.0	NS	0.0	NS	0.0	NS	0.0	NS
**SR**	3	55.4	***	0.48	NS	1.0	NS	21.6	***	14.8	***	2.79	*	1.49	NS	0.31	NS
**Y × SR**	3	0.0	NS	0.0	NS	0.0	NS	14.4	***	0.0	NS	0.0	NS	0.0	NS	0.0	NS
**Cv × SR**	3	11.1	***	0.76	NS	0.94	NS	2.33	NS	12.7	***	0.25	NS	0.5	NS	2.95	*
**Y × Cv × SR**	3	0.0	NS	0.0	NS	0.0	NS	1.56		0.0	NS	0.0	NS	0.0	NS	0.0	NS
**RT × SR**	3	3.3	*	3.99	**	4.89	*	6.48	***	18.1	***	0.12	NS	0.6	NS	2.15	NS
**Y × RT × SR**	3	0.0	NS	0.0	NS	0.0	NS	4.34	**	0.0	NS	0.0	NS	0.0	NS	0.0	NS
**C × RT × SR**	3	3.3	*	4.09	**	7.08	***	6.57	***	11.08	***	0.74	NS	1.65	NS	2.21	NS
**Y × Cv × RT × SR**	3	0.0	NS	0.0	NS	0.0	NS	4.4	**	0.0	NS	0.0	NS	0.0	NS	0.0	NS
**Residual**	3	7.9		0.036		4.5		5.7		0.03		13.7		0.08		4.73	

The experiment was conducted in 2009 and 2010 in Stoneville, MS, USA.

^a^ *Significant at *P* ≤ 0.05; **Significant at *P* ≤ 0.01; ***Significant at *P* ≤ 0.001.

^‡^NS, not significant. Suc = sucrose, raff = raffinose, stac = stachyose, glu = glucose, fruc = fructose.

**Table 4:** Analysis of variance (*F* and *P* values) of the effects of year (Y), cultivar (Cv), row-type (RT, either single- or twin-rows), and seeding rate (SR) on the concentrations of seed sugars (sucrose, raffinose, stachyose, glucose, and fructose, mg g^-1^) and boron (B, mg kg^-1^), phosphorus (P, g kg^-1^), and iron (Fe, mg kg^-1^) in soybean cultivars 94M80 and GP-533 in Beulah fine sandy loam soil.^a^

		Suc		Raff		Stac		Glu		Fruc		B		P		Fe	
Effect	DF	*F*	*P*	*F*	*P*	*F*	*P*	*F*	*P*	*F*	*P*	*F*	*P*	*F*	*P*	*F*	*P*
**Y**	2	348	***	502	***	74.9	***	3.16	NS	33.9	***	50.1	***	81.6	***	51.4	***
**Cv**	1	0.4	NS	69.2	***	201	***	160	***	95.8	***	174	***	941	***	377	***
**Y × Cv**	2	151	***	511	***	63.1	***	2.5	NS	9.75	***	35.0	***	40.4	***	28.5	***
**RT**	1	75.35	***	1.26	NS	0.93	NS	95.0	***	7.07	**	4.53	*	555	***	27.6	***
**Y × RT**	2	0.25	NS	0.12	NS	0.25	NS	23.4	***	0.92	NS	3.06	NS	238	***	8.31	**
**Cv × RT**	1	9.16	*	0.79	NS	0.3	NS	0.36	NS	0.49	NS	3.37	NS	0.00	NS	5.23	*
**Y × Cv × RT**	2	16.9	***	0.34	NS	0.21	NS	11.4	***	0.18	NS	6.45	*	80.5	***	30.4	***
**SR**	3	10.1	***	0.5	NS	0.2	NS	12.3	***	3.87	**	11.5	***	9.68	***	2.4	NS
**Y × SR**	6	7.23	***	0.31	NS	0.85	NS	1.34	NS	3.02	**	8.5	***	7.38	***	1.41	NS
**Cv × SR**	3	6.17	***	0.37	NS	0.09	NS	0.38	NS	0.51	NS	4.3	**	1.67	NS	2.64	*
**Y × Cv × SR**	6	3.69	**	1.55	NS	0.43	NS	1.25	NS	1.01	NS	0.07	NS	2.02	NS	2.45	*
**RT × SR**	3	3.58	*	0.73	NS	0.77	NS	0.15	NS	2.17	NS	4.79	**	8.4	***	0.85	NS
**Y × RT × SR**	6	1.97	NS	0.71	NS	1.02	NS	0.13	NS	1.59	NS	0.78	NS	13.14	***	2.5	NS
**C × RT × SR**	3	0.24	NS	0.98	NS	0.5	NS	0.23	NS	2.7	*	0.82	NS	2.13	NS	1.19	NS
**Y × Cv × RT × SR**	6	0.38	NS	0.69	NS	0.44	NS	0.29	NS	0.45	NS	1.75	NS	3.68	*	0.02	NS
**Residual**		9.1		0.052		6.6		0.07		0.015		22.6		0.081		15.57	

The experiment was conducted in 2008, 2009, and 2010 in Stoneville, MS, USA.

^a^ *Significant at *P* ≤ 0.05; **Significant at *P* ≤ 0.01; ***Significant at *P* ≤ 0.001.

^‡^NS, not significant. Suc = sucrose, raff = raffinose, stac = stachyose, glu = glucose, fruc = fructose.

### Effects of row-type and seeding rate on seed nutrition constituents in soybean cultivars

In clay soil in 2009, the mean values (Table 5) in cultivar 94M80 showed that protein, sucrose, glucose, and B concentrations increased with increasing SR on both row-types. When the maximum concentration was achieved at the higher SR (40 seed m^-2^ or 50 seed m^-2^), however, the concentration became either constant or declined. Linolenic acid concentration in 94M80 was higher on twin-rows than on single rows. For the GP 533 cultivar, mean values showed that protein, palmitic, linolenic, sucrose, and B concentrations increased with increasing SR on both row-types. The concentrations of P increased on single rows only. In 2010, sucrose, glucose, fructose, and B concentrations increased with increasing SR in 94M80, but this increase became constant or declined at the highest seeding rates (40 seed m^-2^ or 50 seed m^-2^), following the same trend as in 2009. Protein, oleic and linolenic acids, sucrose, glucose, and B concentrations showed an increasing trend with increasing SR for GP 533 on single row only. Protein and linolenic acid concentrations were higher in GP 533 than in 94M80 on both row- types, but oil and oleic acid concentrations were lower in GP 533 than in 94M80. Oleic acid was the highest in 2010 in 94M80, but linolenic acid concentration was lower in 94M80 than in GP 533.

**Table 5:** Effects of row-type (single, S, or twin, T) and seeding rate (SR, seed m^-2^) on seed protein, oil, fatty acids (g kg^-1^), sucrose (Suc), raffinose (Raff), stachyose (Stac), glucose (Glu), fructose (Fruc) (mg g^-1^), boron (B, mg kg^-1^), phosphorus (g kg^-1^), and iron (Fe, mg kg^-1^) in two soybean cultivars (94M80 and GP 533) in Sharkey clay soil.

									**2009**	**94M80**						
	**SR**	**Protein**	**Oil**	**Palmitic**	**Stearic**	**Oleic**	**Linoleic**	**Linolenic**	**Suc**	**Raff**	**Stac**	**Glu**	**Fruc**	**B**	**P**	**Fe**
**S**	20	418	229	102	44.8	275	524	59.9	9.3	7.5	45.7	15.8	0.84	25.6	2.9	48.9
	30	429	233	105	44.3	274	523	57.5	11.9	7.6	44.5	23.5	0.79	26.3	2.8	45.5
	40	417	228	105	43.4	272	525	55.7	16.2	7.3	47.3	29.0	0.79	26.8	2.8	46.7
	50	415	229	106	45.3	279	509	59.6	12.0	7.5	45.4	12.8	0.86	26.7	2.9	45.5
	LSD	2.4	2.79	3.98	0.69	2.84	5.19	3.20	1.24	0.10	1.00	2.00	0.05	1.08	0.16	1.56
**T**	20	413	245	107	44.3	283	502	72.2	7.6	7.6	43.7	15.8	0.82	33.3	3.6	56.4
	30	424	243	110	43.9	279	499	73.3	15.0	7.5	44.7	21.5	0.86	36.6	3.6	56.8
	40	437	248	110	43.9	279	499	77.5	11.9	7.6	46.7	21.8	1.4	34.0	3.6	55.2
	50	418	247	106	44.7	284	484	84.7	14.2	7.4	47.0	23.5	1.3	33.7	3.7	56.5
	LSD	2.91	3.25	3.14	0.67	5.19	8.78	6.42	3.69	0.12	1.11	2.04	0.08	1.10	0.11	0.94
									**2009**	**GP533**						
**S**	20	425	210	103	45.3	234	510	98.1	13.3	5.5	28.6	20.3	1.0	39.5	2.8	54.7
	30	428	209	108	45.7	234	510	107.3	17.5	5.4	29.7	26.5	1.0	43.0	2.9	55.9
	40	435	209	115	46.1	233	504	107.6	23.0	5.4	29.2	26.0	1.0	39.7	3.0	55.7
	50	427	211	114	43.7	234	502	111.7	23.3	5.3	31.4	24.8	1.0	39.8	3.0	55.0
	LSD	2.34	1.53	2.27	0.69	2.90	5.21	3.92	1.18	0.08	1.3	0.67	0.04	2.94	0.07	0.85
**T**	20	427	219	103	44.9	226	514	100.1	17.5	5.3	33.1	26.8	1.3	42.3	4.6	64.5
	30	428	218	118	46.1	231	504	104.6	22.9	5.3	30.9	31.0	1.6	45.0	4.3	64.2
	40	446	220	115	44.4	227	504	110.3	29.0	5.4	29.5	35.3	1.7	43.3	4.3	65.7
	50	436	220	112	44.0	228	516	106.8	29.0	5.5	29.8	32.8	1.1	43.2	4.6	66.7
	LSD	3.66	2.50	4.91	0.72	2.20	6.57	2.36	2.06	0.06	0.98	1.93	0.12	1.73	0.20	0.91
									**2010**	**94M80**						
**S**	20	433	207	103	40.9	303	498	54.5	9.3	7.5	45.7	1.6	0.83	25.6	2.9	48.9
	30	431	208	98	41.6	308	501	63.8	11.9	7.6	44.5	2.4	0.79	26.3	2.8	45.5
	40	427	206	105	40.9	307	497	54.2	16.2	7.3	47.3	2.9	0.79	26.8	2.8	46.7
	50	432	207	98	41.3	300	493	60.0	12.0	7.5	45.4	1.3	0.86	26.7	2.9	45.5
	LSD	2.49	1.67	1.87	0.28	6.41	4.50	2.60	1.20	0.10	0.97	0.20	0.05	1.10	0.16	1.60
**T**	20	437	228	103	41.8	300	503	57.1	7.6	7.6	44.7	1.6	0.82	33.3	3.6	56.4
	30	441	220	104	41.0	300	504	56.3	15.0	7.5	47.0	2.2	0.86	36.6	3.6	56.8
	40	436	223	107	42.3	297	506	61.7	11.9	7.6	43.7	2.2	1.43	34.0	3.6	55.2
	50	440	220	102	41.4	308	508	56.9	14.2	7.4	46.7	2.4	1.3	33.7	3.7	56.5
	LSD	2.60	1.50	1.70	0.34	5.70	8.70	2.40	3.70	0.12	1.10	0.20	0.08	1.10	0.11	0.94
									**2010**	**GP533**						
**S**	20	449	181	113	40.1	246	520	64.9	13.3	5.5	28.6	2.0	1.04	39.5	2.8	54.7
	30	453	178	113	40.3	254	518	68.0	17.5	5.4	29.7	2.7	1.00	43.0	3.0	55.9
	40	450	179	113	40.8	253	527	68.5	23.0	5.4	29.2	2.6	1.03	39.7	2.9	55.7
	50	450	177	110	40.1	255	527	72.2	23.3	5.3	31.4	2.5	1.03	39.8	3.0	55.0
	LSD	1.40	2.30	1.90	0.25	6.50	2.70	2.70	1.18	0.08	1.10	0.07	0.04	2.90	0.07	0.85
**T**	20	453	184	113	40.0	258	520	68.4	17.5	5.3	33.1	2.7	1.28	42.3	4.6	64.5
	30	453	182	113	39.4	253	528	70.0	22.9	5.3	30.9	3.1	1.56	45.0	4.3	64.2
	40	451	186	114	40.1	252	527	66.9	29.0	5.4	29.5	3.5	1.70	43.3	4.3	65.7
	50	450	185	115	40.4	254	524	65.0	29.0	5.5	29.8	3.3	1.14	43.2	4.6	66.7
	LSD	1.90	1.40	1.60	0.38	6.30	4.60	3.80	2.10	0.06	1.00	0.19	0.12	1.70	0.20	0.91

The experiment was conducted in 2009 and 2010 in Stoneville, MS, USA.

In the sandy soil in 2008, the mean values in 94M80 cultivar (Table 6) showed that increasing SR resulted in higher protein, sugars (sucrose, glucose, and fructose), and mineral concentrations (B, and Fe) on both row-types, but for oil, palmitic, stearic, raffinose, and stachyose concentrations, there were either stable or inconsistent trend. The increase of seed constituents with SR increased until a maximum level was achieved, then either the concentration decreased or remained constant, following the same pattern as in the clay soil. This pattern was observed among many, but not all, of the seed composition constituents. Twin-rows resulted in higher oil, sugars (sucrose, glucose, and fructose), and minerals (B, P, and Fe) than in single-row. The response of the remainder of the seed constituents was either constant or inconsistent. In the GP 533 cultivar, no obvious or clear trends were observed between seed constituents and SR increases on single-rows. However, on twin-rows, SR increases resulted in higher protein, oleic, glucose, and B and P concentrations. There was no clear pattern observed in the remainder of the seed constituents. Generally, cultivar GP 533 accumulated more seed constituents than 94M80 on both single- and twin-rows, however, cultivar 94M80 accumulated more oil than GP 533 on single and twin rows. In 2009, similar results were observed for protein, sucrose, glucose, fructose, and B concentrations. Cultivar GP 533 accumulated higher levels of seed constituents than 94M80 on both row-types, but cultivar 94M80 accumulated more oil than GP 533, confirming what was observed in 2008. In 2010, there were no clear responses to SR increase on single rows in both cultivars, perhaps due to drier year and high heat in 2010. However, on twin-rows, SR increases resulted in higher seed protein, glucose, and fructose in both cultivars. Oil concentration was higher in 94M80 than in GP-533, in contrast to the concentration pattern of protein.

**Table 6:** Effects of row-type (single, S, or twin, T) and seeding rate (SR, seed m^-2^) on seed protein, oil, fatty acids (g kg^-1^), sucrose (Suc), raffinose (Raff), stachyose (Stac), glucose (Glu), fructose (Fruc) (mg g^-1^), boron (B, mg kg^-1^), phosphorus (g kg^-1^), and iron (Fe, mg kg^-1^) in two soybean cultivars (94M80 and GP-533) in Beulah fine sandy loam soil.

						**2008**		**94M80**								
**Row**	**Rate**	**Protein**	**Oil**	**Palmitic**	**Stearic**	**Oleic**	**Linoleic**	**Linolenic**	**Suc**	**Raff**	**Stac**	**Glu**	**Fruc**	**B**	**P**	**Fe**
	20	412	228	110	40	255	524	68.0	13.7	5.2	30.8	1.3	0.47	45.1	3.0	43.0
	30	432	219	111	39	261	523	71.4	20.0	5.2	31.2	1.6	0.67	54.1	3.2	47.7
**S**	40	435	222	105	39	278	507	79.9	23.4	5.1	31.7	1.6	0.67	65.9	3.0	46.3
	50	437	217	107	39	256	519	83.5	20.2	5.1	31.4	1.7	0.54	67.2	3.3	47.6
	LSD	2.47	2.30	1.47	0.27	5.86	7.47	2.45	1.87	0.04	0.55	0.06	0.01	0.94	0.14	1.92
	20	427	226	108	39	247	534	73.1	18.3	5.2	32.7	2.1	0.66	61.8	4.1	56.2
	30	435	231	110	39	262	521	68.6	25.8	5.2	32.0	2.5	0.69	64.9	4.6	56.6
**T**	40	437	232	111	40	269	517	74.1	27.3	5.1	31.8	2.5	0.75	66.4	4.5	55.3
	50	429	233	109	40	247	525	84.0	21.3	5.2	31.2	2.5	0.66	67.0	5.5	61.1
	LSD	1.45	3.99	2.44	0.27	7.90	6.97	2.50	1.13	0.05	0.55	0.06	0.28	3.07	0.13	1.75
						**2008**		**GP 533**								
	20	465	205	106	40	232	538	92.5	27.2	5.2	32.1	2.2	0.69	56.2	3.6	72.2
	30	464	205	109	40	239	532	85.0	28.4	5.1	32.2	2.3	0.67	65.5	4.1	69.4
**S**	40	463	207	105	40	245	530	83.9	27.2	5.2	32.1	2.3	0.75	66.9	3.5	68.6
	50	466	206	104	40	230	544	86.5	26.8	5.2	32.2	2.3	0.70	68.8	3.5	65.2
	LSD	2.03	0.99	1.83	0.28	4.61	6.10	3.83	0.93	0.06	0.05	0.07	0.02	1.84	0.16	1.60
	20	464	207	105	40	229	544	87.5	32.8	5.3	31.9	2.7	0.75	57.9	5.4	70.3
**T**	30	464	204	110	40	244	525	90.5	38.0	5.2	31.8	2.7	0.78	65.1	5.6	67.5
	40	471	208	108	41	243	527	92.8	29.8	5.4	31.8	2.8	0.76	65.5	6.6	69.4
	50	466	207	107	40	230	544	87.8	28.9	5.2	32.1	2.8	0.73	67.3	6.8	69.1
	LSD	4.05	1.71	2.69	0.82	6.72	6.32	3.09	2.41	0.11	0.71	0.06	0.01	2.30	0.14	2.05
						**2009**		**94M80**								
**Row**	**Rate**	**Protein**	**Oil**	**Palmitic**	**Stearic**	**Oleic**	**Linoleic**	**Linolenic**	**Suc**	**Raff**	**Stac**	**Glu**	**Fruc**	**B**	**P**	**Fe**
	20	415	218	100	44	245	522	101	14.3	5.4	32.3	1.6	0.69	46.8	2.7	64.2
	30	421	218	107	44	217	548	99	17.6	5.4	33.7	1.9	0.76	44.0	2.7	59.8
**S**	40	427	225	111	46	219	528	109	15.0	5.5	32.1	2.1	0.82	52.0	2.7	64.3
	50	413	222	121	45	198	529	113	25.2	5.5	33.5	2.0	0.84	53.0	2.7	64.5
	LSD	2.82	2.38	3.72	0.61	6.78	9.61	3.56	2.69	0.09	1.46	0.12	0.07	1.93	0.27	1.86
	20	430	215	97	45	233	535	95	22.6	5.5	32.4	1.6	0.78	46.2	3.5	62.5
	30	439	228	106	45	223	530	104	27.2	5.5	33.4	1.9	0.81	47.8	3.6	63.4
**T**	40	443	203	111	45	221	534	106	27.0	5.6	33.8	2.2	0.82	50.0	3.6	62.1
	50	445	228	119	44	195	531	117	30.2	5.4	34.9	2.3	0.86	50.8	3.5	62.4
		2.64	2.98	5.13	0.93	10.10	12.30	4.14	1.42	0.07	1.02	0.19	0.05	2.53	0.22	1.85
						**2009**		**GP 533**								
	20	450	199	116	44	208	536	97	10.6	6.3	22.1	2.1	0.97	62.8	4.9	72.7
	30	451	195	114	43	207	540	98	11.8	6.5	22.3	2.4	0.92	64.6	4.9	62.1
**S**	40	460	191	127	46	206	525	105	10.5	6.3	25.1	2.5	1.23	67.1	5.0	68.2
	50	467	200	122	43	199	534	107	14.8	6.4	25.6	2.5	1.12	65.6	5.0	68.9
	LSD	3.60	1.53	3.78	0.76	5.64	5.81	3.14	1.59	0.23	1.10	0.13	0.06	3.85	0.19	2.79
	20	470	195	119	44	212	534	93	11.3	6.7	24.0	2.5	1.01	67.0	5.0	71.4
	30	449	193	120	45	206	527	104	12.6	6.3	22.9	2.7	1.22	68.1	5.0	69.4
**T**	40	459	187	121	45	187	546	107	14.3	6.3	25.0	2.9	1.04	64.5	4.8	73.4
	50	455	187	125	44	185	544	106	13.8	6.4	25.7	2.9	1.09	64.1	4.9	73.3
	LSD	4.38	3.40	3.85	0.66	3.15	7.30	2.03	3.69	0.18	0.89	0.14	0.07	4.20	0.17	2.13
						**2010**		**94M80**								
**Row**	**Rate**	**Protein**	**Oil**	**Palmitic**	**Stearic**	**Oleic**	**Linoleic**	**Linolenic**	**Suc**	**Raff**	**Stac**	**Glu**	**Fruc**	**B**	**P**	**Fe**
	20	421	224	103	42	295	503	61	35.0	7.5	39.5	1.8	0.83	40.4	2.4	53.9
	30	421	225	102	43	301	505	53	32.5	7.4	38.3	1.4	0.85	42.5	2.2	52.3
**S**	40	417	229	102	43	295	510	52	33.5	7.7	44.3	1.4	0.90	40.9	2.4	54.2
	50	420	225	99	43	297	524	48	34.5	7.6	45.0	1.4	0.98	40.8	2.5	55.2
	LSD	2.58	1.80	1.90	0.64	6.00	5.90	3.50	0.69	0.10	1.60	0.16	0.09	2.60	0.13	1.70
	20	413	226	100	42	295	516	53	34.0	7.5	43.3	2.2	1.05	41.4	2.2	48.9
	30	422	223	104	43	301	498	61	34.8	7.7	42.0	2.3	1.14	45.5	2.5	54.7
**T**	40	432	224	102	42	306	499	55	33.3	7.4	40.3	2.4	1.19	42.0	2.3	56.5
	50	435	223	101	42	302	515	58	34.3	7.8	39.8	2.4	1.29	42.8	2.6	59.8
	LSD	3.50	2.40	1.40	0.45	10.10	10.60	3.20	0.63	0.07	2.00	0.15	0.13	2.60	0.15	1.80
						**2010**		**GP 533**								
	20	429	191	117	40	222	552	82	35.0	5.6	33.5	1.7	1.07	56.3	3.4	62.1
	30	429	190	114	40	226	536	83	35.3	5.6	34.8	2.0	1.04	50.8	3.4	63.2
**S**	40	429	189	116	41	228	541	76	34.0	5.6	35.5	1.9	0.99	49.8	3.3	61.1
	50	429	189	117	41	224	536	82	34.5	5.8	31.5	2.2	1.07	50.3	3.3	66.7
	LSD	2.80	1.80	2.80	0.51	7.50	7.80	2.50	0.63	0.12	1.50	0.21	0.15	1.20	0.07	1.90
	20	431	190	113	40	234	531	85	34.3	5.5	33.3	2.3	1.23	53.2	3.4	65.7
	30	433	190	114	40	226	540	80	34.0	5.5	35.0	2.5	1.45	48.7	3.3	64.7
**T**	40	449	190	112	41	241	529	81	36.3	5.6	34.5	2.8	1.43	49.5	3.5	65.7
	50	450	192	116	40	219	534	87	34.8	5.5	33.3	2.7	1.53	50.5	3.4	66.9
	LSD	2.30	1.50	1.70	0.40	5.50	7.40	4.80	0.61	0.11	1.60	0.12	0.05	0.78	0.07	1.20

The experiment was conducted in 2008, 2009, and 2010 in Stoneville, MS, USA.

### Correlations between seeding rates and seed composition in soybean cultivars

In the clay soil and in cultivar 94M80, there were consistent significant (P≤0.05) positive correlations between SR increases and sucrose, glucose, and fructose in 2009 and 2010 on twin-row only. In GP 533, there were significant (P≤0.05) positive correlations between SR and linolenic acid on both row-types in 2009 (Table 7). A consistent significant positive correlation between SR and sucrose and between SR and glucose was observed in GP 533 on both row-types in 2009 and 2010 (Table 7). A significant positive correlation between SR and raffinose, and significant negative correlation between SR and stachyose was also observed in GP 533 in 2009 and 2010, but only on twin-rows (Table 7). In the sandy soil in 94M80, a significant positive correlation was observed between SR and linolenic acid (P value ranged from 0.01 to 0.002) on single rows in 2008 and on both rows in 2009, between SR and sucrose (P value ranged from 0.03 to 0.004) on single-rows, and between SR and glucose (P value ranged from 0.04 to 0.001) on both rows in 2008 and 2009 (Table 8). Boron concentration showed a positive (P value ranged from 0.02 to <0.0001) correlation with SR on single-row only in 94M80 in 2008 and 2009. No consistent correlations were observed between SR and other seed constituents in 94M80 (Table 8).

**Table 7:** Correlations (*R*-values and *P*-values) between seeding rate (SR) and seed composition constituents (protein, oil, fatty acids, and sugars) and minerals (phosphorus, P, and boron, B) in soybean cultivars grown on single- (S) and twin- (T) rows in Sharkey clay soil 2009 and 2010 at Stoneville, MS, USA.^a^

				**2009**	**94M80**						
**RT**	***R* and *P***	**Protein**	**Oil**	**Linolenic**	**Suc**	**Raff**	**Stac**	**Glu**	**Fruc**	**P**	**Fe**
**S**	***R***	-0.34	-0.14	-0.05	0.42	-0.20	0.10	-0.05	0.07	0.01	-0.33
	***P***	NS	NS	NS	NS	NS	NS	NS	NS	NS	NS
**T**	***R***	0.29	0.18	0.38	0.54	-0.17	0.13	0.57	0.75	0.16	-0.07
	***P***	NS	NS	NS	*	NS	NS	*	*	NS	NS
				**2009**	**GP 533**						
**S**	***R***	0.22	0.02	0.54	0.86	-0.48	0.40	0.53	0.01	0.49	0.06
	***P***	NS	NS	*	***	NS	NS	*	NS	NS	NS
**T**	***R***	0.52	0.12	0.52	0.76	0.59	-0.56	0.54	-0.11	0.01	0.47
	***P***	*	NS	*	***	*	*	*	NS	NS	NS
				**2010**	**94M80**						
**S**	***R***	-0.19	-0.06	0.13	0.42	-0.20	0.10	-0.05	0.07	0.01	-0.33
	***P***	NS	NS	NS	NS	NS	NS	NS	NS	NS	NS
**T**	***R***	0.07	-0.54	0.12	0.54	-0.17	0.13	0.57	0.75	0.16	-0.07
	***P***	NS	*	NS	*	NS	NS	*	***	NS	NS
				**2010**	**GP 533**						
**S**	***R***	-0.07	-0.23	-0.33	0.86	-0.48	0.40	0.53	0.01	0.49	0.06
	***P***	NS	NS	NS	***	NS	NS	*	NS	NS	NS
**T**	***R***	-0.33	0.36	-0.21	0.76	0.59	-0.56	0.54	-0.11	0.01	0.47
	***P***	NS	NS	NS	***	*	*	*	NS	NS	NS

^a^ Level of significance was P≤0.05. Suc = sucrose, raff = raffinose, stac = stachyose, glu = glucose, fruc = fructose.

**Table 8:** Correlations (*R*-values and *P*-values) between seeding rate (SR) and seed composition constituents (protein, oil, fatty acids, and sugars) and minerals (boron, B, phosphorus, P, and iron, Fe) in soybean cultivars grown on single- (S) and twin- (T) rows in Beulah fine sandy loam soil in soybean cultivars in 2008, 2009, and 2010 at Stoneville, MS, USA.^a^

				**2008**	**94M80**					
**RT**	***R* and *P***	**Protein**	**Oil**	**Oleic**	**Linolenic**	**Suc**	**Glu**	**B**	**P**	**Fe**
**S**	***R***	0.79	-0.54	0.16	0.81	0.53	0.73	0.95	0.32	0.36
	***P***	***	*	NS	***	*	***	***	NS	NS
**T**	***R***	0.19	0.31	0.04	0.60	0.28	0.70	0.31	0.83	0.39
	***P***	NS	NS	NS	NS	NS	**	NS	***	NS
				**2008**	**GP 533**					
**S**	***R***	0.05	0.28	0.00	-0.29	-0.16	0.27	0.68	-0.26	-0.65
	***P***	NS	NS	NS	NS	NS	NS	**	NS	*
**T**	***R***	0.18	0.13	0.03	0.06	-0.40	0.39	0.59	0.89	-0.05
	***P***	NS	NS	NS	NS	NS	NS	*	***	NS
				**2009**	**94M80**					
**S**	***R***	0.02	0.44	-0.76	0.61	0.52	0.53	0.59	0.04	0.15
	***P***	NS	NS	***	*	*	*	*	NS	NS
**T**	***R***	0.73	0.13	-0.57	0.70	0.68	0.59	0.37	-0.01	-0.06
	***P***	**	NS	*	**	**	*	NS	NS	NS
				**2009**	**GP 533**					
**S**	***R***	0.72	-0.08	-0.27	0.58	0.38	0.57	0.18	0.14	-0.10
	***P***	0.00	NS	NS	*	NS	*	NS	NS	NS
**T**	***R***	-0.37	-0.45	-0.87	0.70	0.29	0.49	-0.18	-0.11	0.27
	***P***	0.16	NS	***	**	NS	NS	NS	NS	NS
				**2010**	**94M80**					
**S**	***R***	0.16	-0.21	0.18	-0.36	-0.26	-0.22	0.11	-0.05	-0.04
	***P***	NS	NS	NS	NS	NS	NS	NS	NS	NS
**T**	***R***	0.29	-0.23	0.03	0.43	0.35	0.13	0.26	0.43	0.45
	***P***	NS	NS	NS	NS	NS	NS	NS	NS	NS
				**2010**	**GP-533**					
**S**	***R***	-0.03	-0.07	0.01	0.36	0.15	0.40	-0.37	0.05	0.43
	***P***	NS	NS	NS	NS	NS	NS	NS	NS	NS
**T**	***R***	0.08	0.24	-0.58	0.08	-0.32	0.21	-0.40	-0.28	0.03
	***P***	NS	NS	*	NS	NS	NS	NS	NS	NS

^a^ Level of significance was P≤0.05. Suc = sucrose, and glu = glucose.

**Fig 1:**
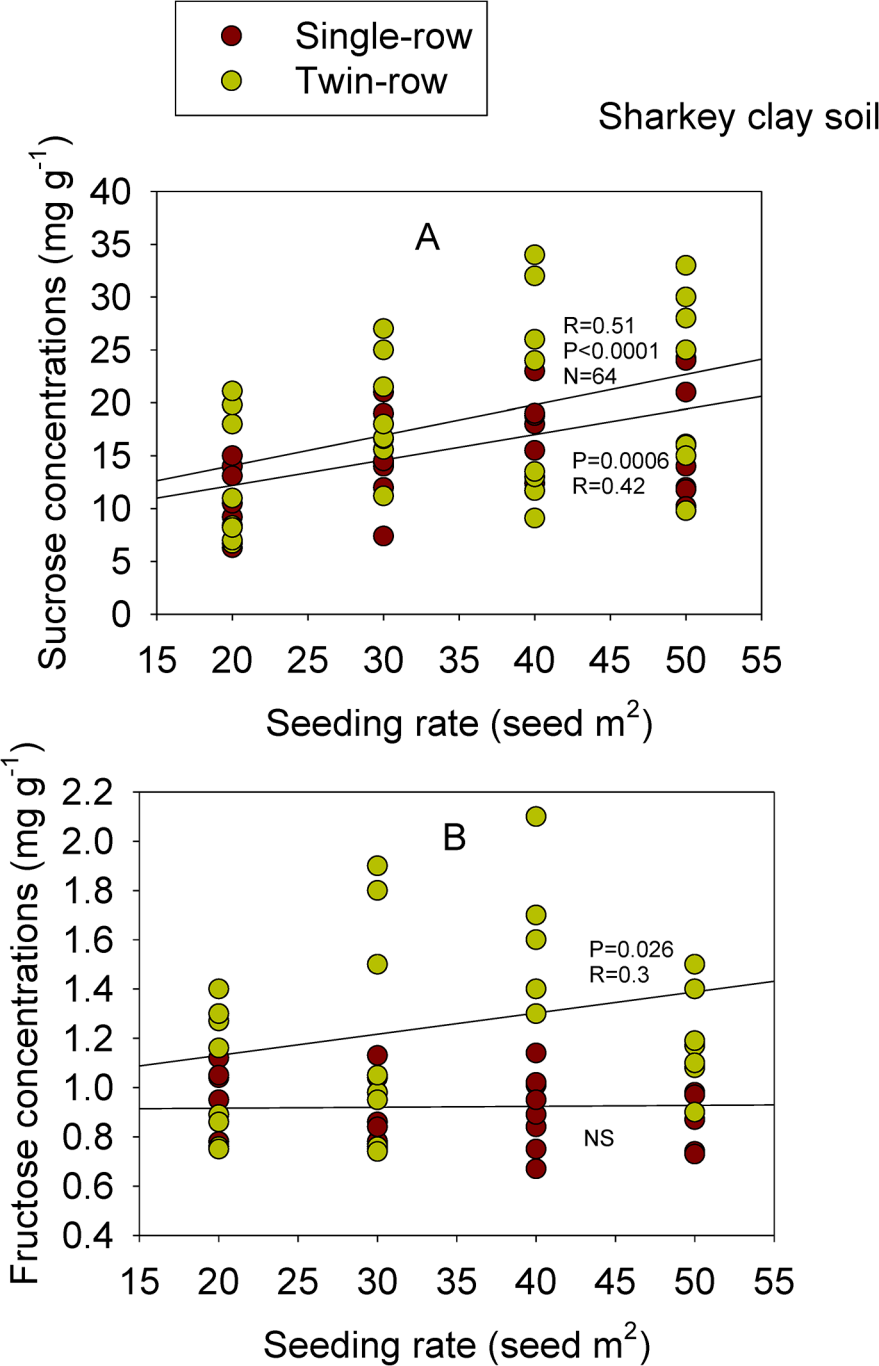
Correlation between seeding rate (SR) and soybean seed sucrose (A) and fructose (B) on single- and twin-rows in Sharkey clay soil across cultivars and row-spacing.

**Fig 2:**
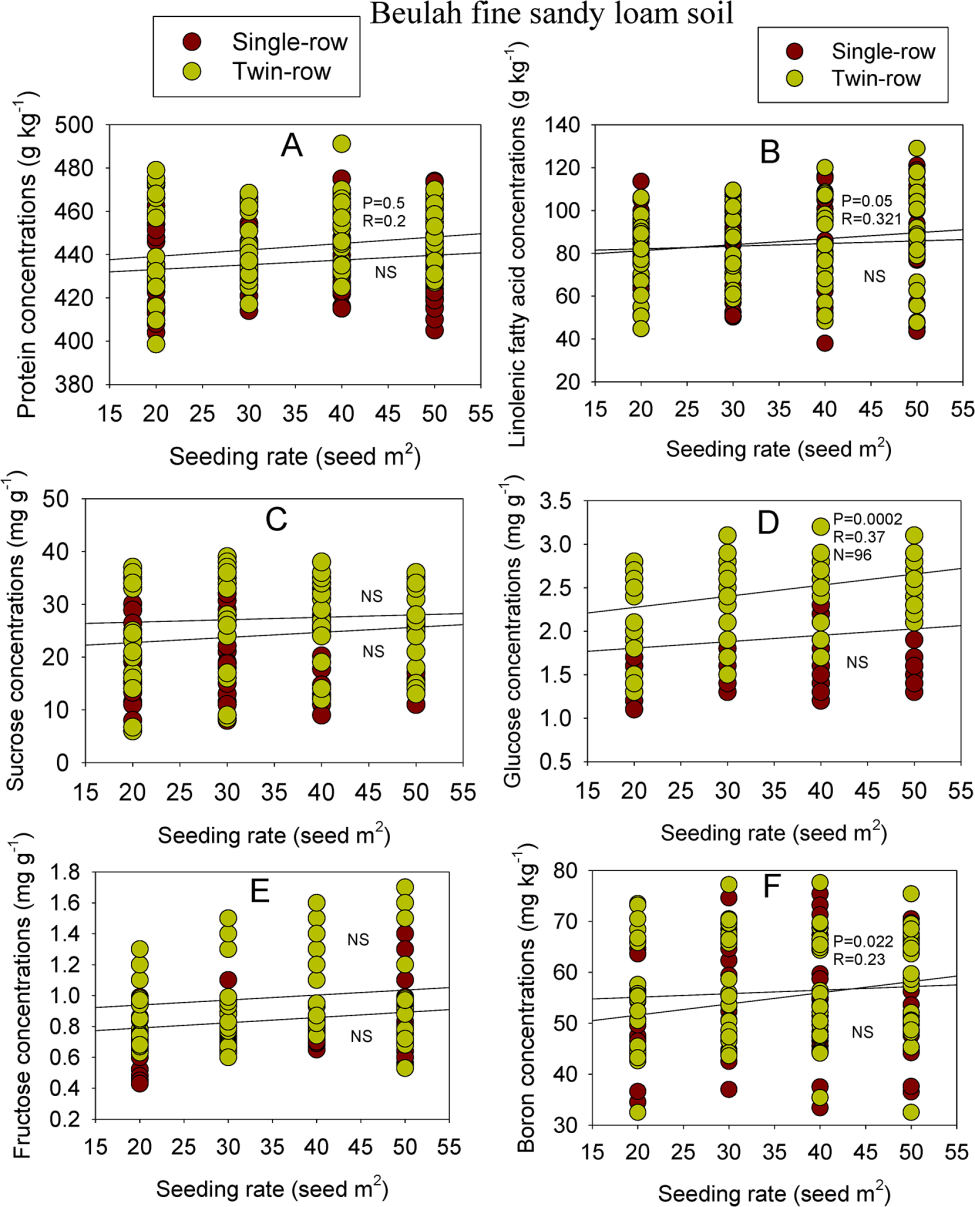
Correlation between seeding rate (SR) and soybean seed protein (A), linolenic fatty acid (B), sucrose (C), glucose (D), fructose (E), and boron 9B0 (F) on single- and twin-rows in sandy loam soil across cultivars and row-spacing.

In GP 533, linolenic acid concentration had a significant (P≤0.01) correlation with SR on single- and twin-rows in 2009 in GP-533. Glucose had a positive correlation with SR on single-row only in GP 533 in 2009. Concentration of B was significantly (P<0.05) correlated with SR in GP 533 on both row-types, but only in 2008. Phosphorus concentration had significant (P<0.0001) correlation with SR on twin-rows only in GP 533 in 2008. There were either no correlations or inconsistent correlations observed between the remainder of the seed constituents and SR on both row-types and in both soils (Table 7 and 8). When correlations were performed across the two cultivars, sucrose and fructose showed significant positive correlation with SR, depending on RT in clay soil (Fig 1). Similar observation was noticed for seed protein, linolenic acid, sucrose, glucose, fructose, and B in sandy soil (Fig 2).

## Discussion

### Effects of seeding rate and row-type on protein, oil, fatty acids, sugars, and minerals

The increasing trend of protein and sugar concentrations in seeds, especially sucrose and glucose, with increased seeding rates (SR) could be due to higher nutrient uptake, transport, and accumulation in seeds. The higher accumulation of these nutrients in seeds could be due to higher light interception, early canopy closure, and a greater rate of photosynthesis assimilates resulted from increased seeding rates [13, 31, 32, 33, 34, 35, 36]. Research on other species such as safflower (*Carthamus tinctorius*), using SR of 60, 50, 40, and 30 plants m^-2^, showed that oil content was highest under irrigated conditions at the lowest population density of 30 plant m^-2^ and that there was no response of oil to SR at 40, 50, and 60 plant m^-2^ [37]. In contrast, others found that increased SR in sunflower (*Helianthus annuus* L) resulted in increased oil concentrations, and this increase occurred from 30,000 to 45,000 plants ha^-1^, with only small increases in oil beyond 45,000 plant ha^-1^ [38]. Our results on soybean showed that, generally, a rate of 40 plants m^-2^ maximized the concentrations of seed protein, sugars, and minerals, and that response of these constituents to rates beyond this rate was minimal. A seeding rate between 30 and 40 plants m^-2^ could be used for maximizing seed protein, sugars, and B and P concentrations. The optimum SR could be different, depending on cultivar, yearly environmental conditions, heat and drought, and soil texture.

Research on row-spacing (RS) effects showed that RS and irrigation significantly influenced protein and oil contents in soybeans seed, and that an RS of 70 cm resulted in the highest protein content, followed, in decreasing order, by RS of 60, 40, and 50 cm [12]. RS had a significant influence on oleic and linoleic acid content, and a RS of 50 cm produced maximum oil. The RS of 70 cm produced the highest protein (391 g/kg^-1^), but RS of 50 cm produced the lowest (377 g/kg^-1^). Working on canola and using RS 30, 40, and 50 cm, it was found that there were no relationships between RS and oil in canola, but found interaction between varieties and RS, and that oil concentration at RS of 30 cm was the highest [15]. The beneficial effects of narrow-row and twin-row planting was explained in terms of a) increased radiation interception and early canopy closure; b) increased leaf area and greater photosynthesis rates; c) higher mineral nutrient uptake, assimilation, and transport; and d) higher water use efficiency. It was observed that there were differences between narrow- and wide-row soybean in radiation interception during R6 (beginning seed-fill) to the R7 (full seed-fill) stages [31], and in leaf area distribution and duration [32]. Row-spacing of 25 cm resulted in about 80% light interception, but only 70% was observed on 100 cm row spacing [32]. It was found that the full canopy (95% light interception) [34, 35, 39, 40] resulted in greater radiation interception, greater photosynthetic rate [33], and possibly greater nutrient uptake, assimilation, and translocation. Therefore, it is possible that the higher seed protein content observed in our experiment could be due to higher leaf area and light interception [41], higher nitrogen metabolism and photosynthesis rates, resulting and higher protein accumulation in seeds. Although the beneficial effects of twin-rows were explained in terms of light interception, early canopy closure, and radiation use efficiency, the radiation use efficiency was found to be related to cultivar, temperature [35], water [42], and nutrient availability [13]. It was also reported that the different responses of protein, oil, and fatty acids to RS were dependent on environmental conditions, including year, temperature, and precipitation [13, 17].

The increase of protein, sucrose, glucose, fructose, and P and B concentrations with SR was not accompanied by increases in these constituents beyond SR of 40 plants m^-2^. Instead, the concentrations of these constituents either remained constant or declined beyond SR of 40 plants m^-2^. This could be due to higher population density and shade effects resulting from higher SR, leading to higher competition of plants for soil moisture and soil nutrients [43, 44, 45]. Other researchers observed similar pattern in other species such as safflower, where SR of 30 plant m^-2^ showed the highest oil levels, but oil at SR of 40, 50, and 60 plant m^-2^ did not show any significant response [37]. Although the mechanisms of mineral nutrient involvement with seed composition constituents are still not well understood, our experiment generally showed that seed protein and sugars, especially sucrose, glucose, and raffinose concentrations were accompanied with an increase in P and B in seed, especially between 20 and 30 plants m^-2^. The possible involvement of minerals with seed composition was previously reported, although consistent results have not been yet established [13, 46, 47, 48].

**Fig 3:**
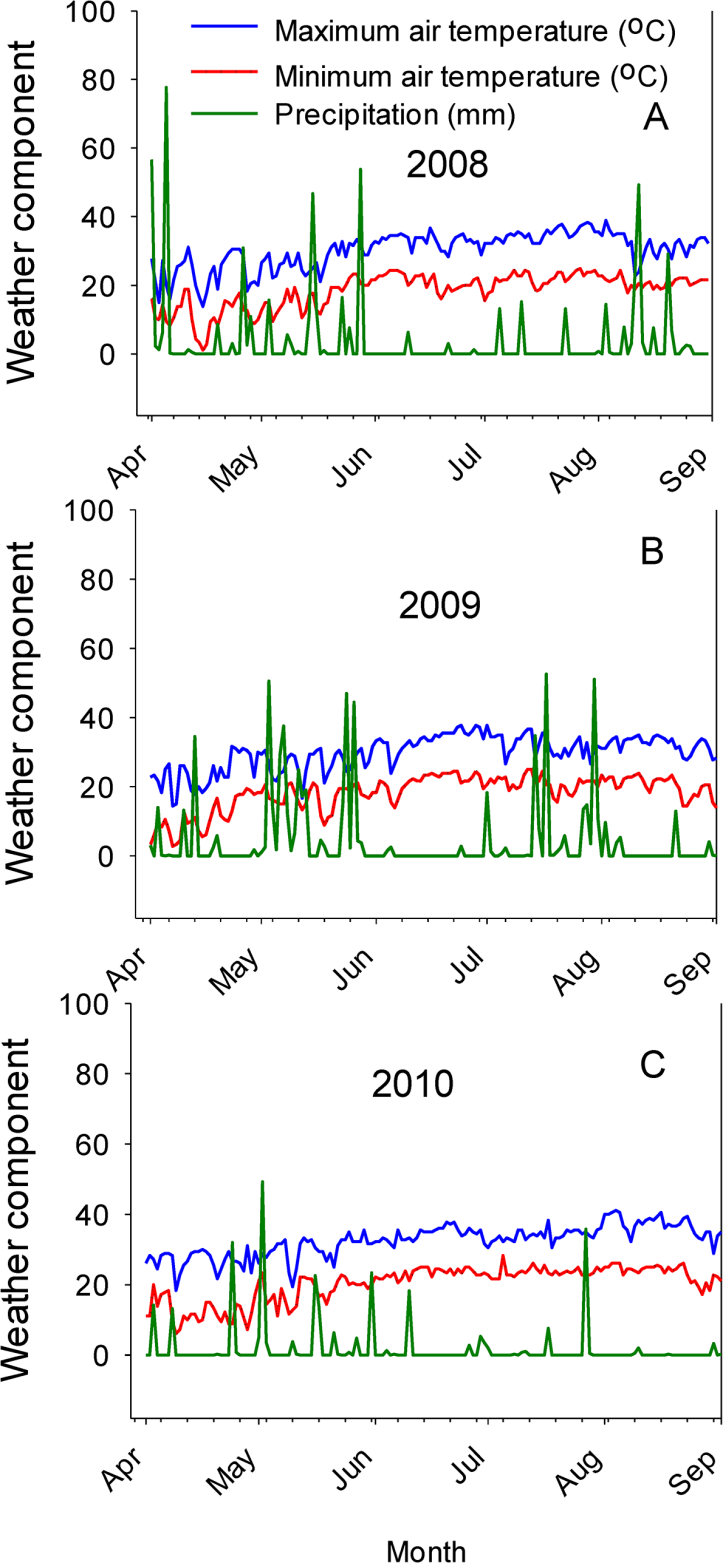
Weather components (minimum and maximum air temperature, and precipitation) in 2008 (A), 2009 (B), and 2010 (C). Weather data obtained from MSUCares (2014).

The increase of linolenic acid concentrations with increasing SR in both cultivars, especially on twin-rows in 2008 and 2009 in 94M80, and in 2009 in GP 533, may be due to shade effects resulting from higher plant density at higher SR. The lack of observation of this pattern in 2010 in both soils and on both row-types could be due to physiological and biochemical disturbances of nutrients synthesis due to growth conditions and environmental factors, including drought and high heat in 2010 (Fig 3). The average maximum temperature reached 34°C in July and 37°C in August in 2010 compared with 32.2°C and 34.4°C in July and August, respectively, in 2009 (Fig 3). The precipitation was 46.0 mm in July and 6.1 mm in August in 2010 compared with 203.7 mm in July and 36.1 mm in August in 2009 (Fig 3). During this period soybeans are normally at seed-fill (R5-R6) stages and nutrient mobility takes place at higher rates, but high heat and drought could reduce the rates of nutrient uptake, assimilation, and transport. The drought and high heat during this period in 2010 may explain the lower levels of minerals in leaves and seed, although adequate levels of nutrients were present in both soils.

The higher accumulation of protein, B, P, and Fe concentrations in GP 533 than in 94M80, especially in the clay soil could be due to soil texture and a longer maturity period of GP 533, allowing for more accumulation of protein and minerals in GP 533. The cultivar GP 533 required 142 days to maturity [9, 20] and 94M80 cultivar required 124 days to maturity [9]. The higher oil concentrations in 94M80 than in GP 533 could be due to the inverse relationship between oil and protein concentrations as GP 533 accumulated higher protein and lower oil concentration than in 94M80. The increase of linolenic acid concentration with increasing SR, especially in the sandy loam soil for 94M80 may be due to shade effects resulting from higher SR and higher plant populations, possibly by creating a cooler micro-environment in the lower canopy. Previous research showed that highly shaded soybean leaves, caused by high plant density, resulted in accumulation of triacylglycerol up to 25% of total leaf lipid compared to leaves in the upper canopy [49]. They also found that shade resulted in lower linolenic acid content, which was accompanied by a proportional increase in oleic and linoleic acids, and concluded that triacylglycerol accumulation was due to altered carbon metabolism. The lower concentrations of B, P, and Fe in 2010 in both soils in 94M80 were due to high heat and drier year in 2010 (Fig 3), while the GP 533 cultivar was less affected by the drought and heat due to a longer duration of maturity, allowing for more mineral accumulation, and cooler temperature towards the end of the growing season under the Mississippi delta climate.

### Correlations between seeding rates and seed composition constituents

The positive correlation between linolenic acid and SR in 94M80 or GP 533 on both row-types in clay or sandy soils in 2009 may be due to increasing SR that resulted in higher population density, creating shade and lowering temperature in the plants' lower canopy. Both shade and lower temperature were reported to increase linolenic acid [49, 50]. This relationship was not observed in 2010 due to higher temperatures and drought as temperature [51] and drought [52] can alter the level and relationship between seed composition constituents. The significant correlation between sucrose and glucose with SR in single- and twin-rows in 2009 and 2010 in clay soil and sandy soil indicated that these sugars were the constituents most affected by seeding rates, and perhaps due to the strong association of these sugars with light radiation interception and the photosynthesis process. It appears that increasing SR resulted in early canopy closure and higher radiation use efficiency and photosynthesis rates [13, 32, 35, 36], resulting in higher transport rates of sucrose and glucose within the plants and higher mobility of these sugars from leaves to seed, especially during the seed-fill stage. The correlation between SR and sugars differed, depending on row-type, indicating that cultivars may respond differently to row-type. Also, the fact that both B and P concentrations were also positively correlated with SR in sandy soils on single rows only for B and twin-rows only for P indicated possible effects of soil texture and row-type on this correlation.

The negative correlation between SR and stachyose may be due to a possible inverse relationship of sucrose, glucose, and fructose with stachyose, especially under environmental stress conditions of high heat and drought as stachyose may have a role in heat and desiccation tolerance and seed protection [13, 53, 54]. Although seed composition constituents such as protein, sucrose, glucose, fructose, and minerals increased with increasing SR on both single and twin rows in sandy and clay soils in 2008 and 2009, a linear correlation between some of these constituents such as protein and SR could not be established because protein concentrations reached maximum levels at seed rates of 20 and 30 seed m^-2^ and then protein either remained constant or declined at the higher SR of 40 or 50 seed m^-2^. The significant positive correlation between SR and sucrose and fructose in sandy soil, and the significant positive correlation between SR and seed protein, linolenic acid, sucrose, glucose, fructose, and B in sandy soil support previous observation that the most affected seed constituents were protein, linolenic fatty acid, sucrose, fructose, and B [13, 19]. This correlation dependent on row-type and soil-type, indicating the significant effects of row-spacing planting and soil nutrients on soybean seed nutritional qualities.

## Conclusions

This research demonstrated that increasing seeding rate resulted in increases of protein, linolenic acid, sucrose, glucose, raffinose, B, and P concentrations on both single- and twin-rows. However, the increase of these constituents became either constant or declined at the higher rates (40 and 50 seeds m^**–2**^). A later maturity cultivar (GP 533) accumulated higher seed protein and linolenic acid than the earlier maturity cultivar (94M80) on both row- types under normal conditions of temperature and drought. However, the earlier maturity cultivar (94M80) accumulated higher oil and oleic acid concentrations in seeds. The positive correlation between sucrose and glucose with SR in single- and twin-rows in 2009 and 2010 in clay soil and sandy soil indicated that these sugars were the most influenced by seeding rates, and the negative correlation between SR and stachyose may be due to a possible inverse relationship between stachyose, and sucrose, glucose, and fructose, especially under environmental stress conditions of heat and drought. Since the positive response of seed protein, sugars, and minerals was between 20 and 40 seed m^-2^, SR beyond 40 seed m^-2^ may have a negative impact on seed quality as they create inter-plant competition for water and nutrients. Since we used limited cultivars in our study, we cannot generalize the trend of seed nutrients obtained in this experiment to be shown by other cultivars unless a big number of cultivars with different maturity groups and genotypes are used. These results are useful to soybean growers for optimizing agronomic practices for higher seed quality. Since higher seed protein and sugars, especially sucrose, glucose, and fructose, are desirable traits for soybean nutrition and taste, optimizing seeding rate to achieve higher levels of these seed constituents is an important goal for soybean industry, and needs further research.
